# Impact of short-term low-dose tamoxifen on molecular breast imaging background parenchymal uptake: a pilot study

**DOI:** 10.1186/s13058-019-1120-5

**Published:** 2019-03-08

**Authors:** Carrie B. Hruska, Katie N. Hunt, Amy Lynn Conners, Jennifer R. Geske, Kathleen R. Brandt, Amy C. Degnim, Celine M. Vachon, Michael K. O’Connor, Deborah J. Rhodes

**Affiliations:** 1 Department of Radiology, Mayo Clinic, 200 First Street SW, Rochester, MN 55905 USA; 2 Department of Health Sciences Research, Mayo Clinic, 200 First Street SW, Rochester, MN 55905 USA; 3 Department of Surgery, Mayo Clinic, 200 First Street SW, Rochester, MN 55905 USA; 4 Department of Medicine, Mayo Clinic, 200 First Street SW, Rochester, MN 55905 USA

**Keywords:** Molecular breast imaging, Tamoxifen, Tc-99m sestamibi, Background parenchymal uptake

## Abstract

**Background:**

High background parenchymal uptake (BPU) on molecular breast imaging (MBI) has been identified as a breast cancer risk factor. We explored the feasibility of offering a short-term intervention of low-dose oral tamoxifen to women with high BPU and examined whether this intervention would reduce BPU.

**Methods:**

Women with a history of high BPU and no breast cancer history were invited to the study. Participants had an MBI exam, followed by 30 days of low-dose oral tamoxifen at either 5 mg or 10 mg/day, and a post-tamoxifen MBI exam. BPU on pre- and post-tamoxifen MBI exams was quantitatively assessed as the ratio of average counts in breast fibroglandular tissue vs. average counts in subcutaneous fat. Pre-tamoxifen and post-tamoxifen BPU were compared with paired *t* tests.

**Results:**

Of 47 women invited, 22 enrolled and 21 completed the study (10 taking 5 mg tamoxifen, 11 taking 10 mg tamoxifen). Mean age was 47.7 years (range 41–56 years). After 30 days low-dose tamoxifen, 8 of 21 women (38%) showed a decline in BPU, defined as a decrease from the pre-tamoxifen MBI of at least 15%; 11 of 21 (52%) had no change in BPU (within ± 15%); 2 of 21 (10%) had an increase in BPU of greater than 15%. Overall, the average post-tamoxifen BPU was not significantly different from pre-tamoxifen BPU (1.34 post vs. 1.43 pre, *p* = 0.11). However, among women taking 10 mg tamoxifen, 5 of 11 (45%) showed a decline in BPU; average BPU was 1.19 post-tamoxifen vs. 1.34 pre-tamoxifen (*p* = 0.005). In women taking 5 mg tamoxifen, 2 of 10 (20%) showed a decline in BPU; average BPU was 1.51 post-tamoxifen vs.1.53 pre-tamoxifen (*p* = 0.99).

**Conclusions:**

Short-term intervention with low-dose tamoxifen may reduce high BPU on MBI for some patients. Our preliminary findings suggest that 10 mg tamoxifen per day may be more effective than 5 mg for inducing declines in BPU within 30 days. Given the variability in BPU response to tamoxifen observed among study participants, future study is warranted to determine if BPU response could predict the effectiveness of tamoxifen for breast cancer risk reduction within an individual.

**Trial registration:**

ClinicalTrials.gov NCT02979301. Registered 01 December 2016.

## Background

Molecular breast imaging (MBI), a nuclear medicine test performed with the radiotracer Tc-99m sestamibi and a dedicated gamma camera, has shown utility for supplemental screening in women with dense breasts on mammography. Because mammography relies on the differences in X-ray attenuation among breast structures to detect cancer, tumors can be masked by surrounding fibroglandular tissue. Fibroglandular tissue of the breast, called breast density when imaged by mammography, has similar X-ray attenuation properties to tumors. In contrast, MBI depicts preferential uptake of Tc-99m sestamibi in metabolically active cells and is able to reveal cancers obscured by density on mammography. In recent studies, the addition of MBI to mammography in women with dense breasts resulted in an additional 7.7 to 8.8 cancers per 1000 women screened [1, 2].

Besides localizing in breast lesions, Tc-99m sestamibi is also taken up to a lesser degree by surrounding non-malignant breast tissue. Background parenchymal uptake (BPU) is the term used to describe the relative uptake in breast fibroglandular tissue to that in subcutaneous fat. BPU is subjectively assessed into categories (photopenic, minimal to mild, moderate, or marked) but can also be measured quantitatively [3, 4]. An analysis of women presenting for screening MBI showed that most (63%) have minimal to mild BPU, which describes a similar or slightly higher level of uptake in fibroglandular tissue and fat; however, 15% of women have moderate or marked BPU, in which higher uptake is seen in fibroglandular tissue compared to fat, and 22% of women have photopenic BPU, in which fibroglandular tissue shows less uptake than in fat [5].

The etiology of BPU is not well-established; however, it may be a global indicator of the functional status of fibroglandular tissue. In breast tumors, Tc-99m sestamibi uptake is influenced by tissue perfusion, angiogenesis, and mitochondrial activity, which is influenced by cellular proliferation and apoptosis [6, 7]. High BPU, assessed both subjectively and quantitatively, has been associated with higher breast cancer risk, independent of mammographic density. In a case-control study, the odds of being diagnosed with an incident breast cancer among women with moderate or marked BPU was three to five times that of women with photopenic or minimal to mild BPU, with a median time to diagnosis of 3.1 years after MBI [8]. In this analysis, the range of BPU categories (from photopenic to marked) was observed among women with similarly dense breasts, suggesting that BPU captures additional information about fibroglandular tissue’s potential for cancer development that is not appreciated by its anatomical appearance on a mammogram. It is not yet known whether changes in BPU, either occurring naturally over time or as a result of interventions, indicate concomitant changes in breast cancer risk.

BPU can be influenced by hormonal factors. Although not an exact relationship, higher BPU is more likely in premenopausal women than in postmenopausal, and in some premenopausal women (29% in a recent study), BPU is higher in the luteal phase of the menstrual cycle compared to the follicular phase [5, 9]. Higher BPU is also associated with postmenopausal hormone therapy [5]. In our practice, withdrawal of postmenopausal hormones has been used as a strategy to reduce BPU in some patients and improve MBI’s ability to distinguish breast lesions. Because of these hormonal associations, we hypothesized that agents which block the action of estrogen in breast tissue, such as tamoxifen, would also decrease BPU, although no prospective studies to date have confirmed this.

Tamoxifen is FDA-approved for reducing recurrence of estrogen receptor-positive breast cancer and also as a chemopreventive agent for women at high risk for breast cancer. The National Surgical Adjuvant Breast and Bowel Project showed preventive oral tamoxifen given to high-risk women, at 20 mg/day for 5 years, reduced breast cancer risk by approximately one half [10]. However, at the standard clinical dose of 20 mg/day, tamoxifen increases the risk of vasomotor symptoms, thromboembolic events and endometrial cancer, which may partially explain low overall utilization of tamoxifen in chemoprevention; recent analyses showed that fewer than 10% of high-risk women offered an anti-estrogen therapy for prevention agree to take it [11]. To address the toxicity concerns, the efficacy of “low-dose” tamoxifen has been investigated. Tamoxifen at 5 mg/day has been proposed as an optimal dose for decreasing the biomarkers of breast disease while mitigating the side effects seen at the 20 mg dose [12–14]. In a study of women being treated for breast cancer, 10 mg tamoxifen per day over a period as short as 14 days resulted in clinically and statistically meaningful declines in tumor molecular markers of estrogen receptor positivity, progesterone receptor positivity, and cellular proliferation marker Ki-67 [15].

While the reduction of tamoxifen’s side effects is important to improve utilization, decision-making about whether to take tamoxifen for chemoprevention could be informed by providing women with reliable information about its effectiveness for their personal risk reduction. Tamoxifen is not universally effective but metabolizes differently among women, where ultrarapid or extensive metabolizers are hypothesized to receive the most benefit from tamoxifen, while intermediate or poor metabolizers receive little benefit [16]. Additionally, imaging technologies may offer personalized monitoring of the effects of tamoxifen at the breast tissue level that may serve as a biomarker for efficacy in reducing breast cancer risk. Declines in mammographic density after 12 to 18 months of chemopreventive tamoxifen have been associated with a lower likelihood of breast cancer diagnosis [17]. In one study of women with BRCA1/2 gene mutations who underwent risk-reducing salpingo-oophorectomy, a subsequent reduction in background parenchymal enhancement (BPE) on breast magnetic resonance (MR) imaging distinguished the group that did not develop breast cancer from the group that did [18]. MR imaging and MBI have both shown potential to provide additional risk information than can be obtained with mammographic density alone [8, 19, 20]. These functional techniques may offer determination of tamoxifen response over a shorter time period as functional changes in fibroglandular tissue are likely to be appreciated more rapidly than anatomic changes seen on mammography.

As a first step toward characterizing the impact of tamoxifen on MBI, we aimed to explore the feasibility of offering a short-term intervention of low-dose oral tamoxifen to women with high BPU and to examine whether this intervention would reduce BPU.

## Methods

In this prospective, single-institution pilot study, healthy volunteers with a history of high BPU were enrolled to undergo a current (pre-tamoxifen) MBI, an intervention of 30 days low-dose tamoxifen, followed by a post-tamoxifen MBI. The enrollment took place between January 2017 and February 2018.

### Recruitment of participants

To identify the potential study participants, we reviewed medical records for women with a history of high BPU, where subjectively assessed categories of moderate or marked BPU were considered high [3]. Women age 40 and older with high BPU on their most recent MBI, performed within 3 years prior to the study enrollment, who appeared to meet the eligibility criteria per medical record review were sent a letter of invitation. The letter informed the recipient that she did have high BPU on her most recent MBI examination. The letter explained that while high BPU is a normal finding, recent research suggests that high BPU may be associated with an increased risk of future breast cancer. Women were encouraged to call the study investigators if they wished to discuss their BPU. The letter also invited women to participate in a research study to investigate if taking tamoxifen could reduce high BPU.

Respondents to the letter were informed of the study procedures, including two MBI exams performed 30 days apart and daily dosages of tamoxifen between the MBI exams. Women were informed of the potential risks of tamoxifen, including common side effects of vasomotor symptoms (such as hot flashes and night sweats), and rare but possible serious risks of tamoxifen, including increased risk of uterine cancer, abnormal blood clots, or vision changes. Women were informed that based on prior studies of low-dose tamoxifen and the short-term use of 30 days in this study, we anticipated a low likelihood of side effects or serious risks [12–15, 21, 22]. Women were informed that they could stop their participation in the study at any time if unwanted side effects occurred. Remuneration was offered for study participation.

### Inclusion/exclusion criteria

Women were eligible to participate if they met the following criteria: (1) no personal history of any type of malignancy; (2) no current breast concerns; (3) no breast implants; (4) not pregnant or lactating; (5) no current or recent use (within 6 months prior to enrollment) of hormonal medications, including systemic estrogen or progesterone hormone therapy, systemic hormonal contraceptives, selective estrogen receptor modulators, aromatase inhibitors, gonadotropin-releasing hormone analogs, prolactin inhibitors, androgens, or antiandrogens; and (6) no current use of drugs known to be strong inhibitors of CYP2D6 which is the major P450 enzyme that metabolizes tamoxifen (bupropion, fluoxetine, paroxetine, quinidine) [23]. To ensure appropriate prescribing of tamoxifen, eligible women also were required to meet the following criteria: (7) no current use of anticoagulants; (8) no history of deep vein thrombosis or pulmonary embolism; (9) no history of transient ischemic attack or cerebrovascular accident; (10) no active proliferative disorders of the endometrium such as atypical hyperplasia, endometriosis, or unresected polyps; (11) no retinal disorders or severe cataract; (12) not currently a smoker; (13) blood pressure below 140/90; and (14) agreed to avoid pregnancy during the study and for 2 months after discontinuing tamoxifen.

Prior to enrollment, a study physician verified the eligibility and absence of documented contraindications to tamoxifen in participants, initially through review of records and information collected during the study screening and consent process. If inadequate information was available for this assessment, participants were interviewed by the study physician.

### Collection of patient characteristics

A questionnaire was administered to the participants in order to obtain information about breast cancer risk factors necessary to calculate 5-year breast cancer risk according to the Gail Model. Mammographic density was obtained from clinical reports of the most recent mammogram prior to enrollment and was assessed according to the Breast Imaging Reporting and Data System (BI-RADS) 5th edition [24]. Participants self-reported their menopausal status. Participants were classified as postmenopausal if they had not had a menstrual period for longer than 12 months and perimenopausal if they reported irregular menstrual cycles that were longer than 35 days or were experiencing menopausal vasomotor symptoms; otherwise, participants were classified as premenopausal. In premenopausal participants, the day of the last menstrual period prior to each MBI examination was recorded in order to calculate the day of the cycle at each imaging time.

### MBI examinations

Each participant underwent two MBI examinations performed in an identical manner. One MBI was performed prior to initiating tamoxifen (pre-tamoxifen MBI), and the second was performed after completing 30 days of tamoxifen (post-tamoxifen MBI). The pre-tamoxifen MBI was targeted to be performed on the day of tamoxifen initiation but allowed to be performed up to 3 days prior. The post-tamoxifen MBI exam was targeted to be performed the day after completing 30 days tamoxifen but was allowed to be performed up to 3 days after finishing tamoxifen. This 30-day period was intentionally selected both to provide a short-term intervention and to enable imaging at a similar time of the menstrual cycle for premenopausal participants, in order to avoid changes in BPU that can occur due to cyclic effects [9].

Radiotracer injections and MBI acquisitions were performed by nuclear medicine technologists with specialized training in mammographic positioning techniques. Prior to injection, participants were asked to fast for a minimum of 3 h in order to improve the uptake of Tc-99m sestamibi in breast tissue by reducing splanchnic and hepatic blood flow [25]. For a few minutes (5 to 10 min) prior to injection, participants were given a warm blanket to wrap around their shoulders and chest, in order to increase peripheral blood flow to the breast tissue and thus improve Tc-99m sestamibi uptake [25]. Participants received an intravenous injection of 8 mCi (300 MBq) Tc-99m sestamibi in an antecubital vein of either arm.

MBI imaging commenced within 5 min after injection. Imaging was performed with a cadmium zinc telluride-based dual-head gamma camera system (LumaGem, CMR Naviscan, Carlsbad, CA) equipped with matched collimators optimized for low-radiation dose imaging. An energy acceptance window of 110–154 keV was used. Bilateral craniocaudal (CC) and mediolateral oblique (MLO) views were acquired for 10 min per view. A prior analysis showed that sestamibi uptake in the breast is rapid and stays consistent throughout the course of the exam [26]. For each view, patients were seated at the system with the breast positioned between the two detectors. Light compression was applied to stabilize the breast and to limit motion.

### Tamoxifen

This study was initially designed to test the impact of 5 mg/day tamoxifen on BPU. However, an interim analysis performed after the first 10 participants completed 5 mg tamoxifen showed a lower than expected number of participants with declines in BPU. Therefore, the protocol was modified to prescribe 10 mg/day tamoxifen in subsequent participants.

Participants were prescribed 30 tamoxifen tablets with strength of either 5 mg or 10 mg. Instructions were to take one tablet per day for 30 days at about the same time each day. Prescriptions were filled through our institution’s research pharmacy service. Tamoxifen (tamoxifen citrate) is available as 10 mg tablets. To obtain 5 mg tablets, 10 mg tablets were split and over-encapsulated by the pharmacy.

Initiation of tamoxifen was targeted to begin on the day of the pre-tamoxifen MBI exam but was allowed to begin up to 3 days after. Throughout the 30-day period, participants were asked to record the day and time of each dose in a drug diary. They were also asked to record any symptoms experienced and the severity of symptom as mild (awareness of sign or symptom; easily tolerated and does not affect ability to perform normal daily activities), moderate (significant discomfort which interfered with the ability to perform normal daily activities), or severe (marked discomfort with an inability to carry out normal daily activities). Participants were contacted by phone at 5 and 20 days after the initiation of tamoxifen to assess for any worrisome symptoms that would preclude further study participation. Compliance was assessed by review of drug diaries and pill count at the end of the 30-day period.

### Quantitative BPU assessment

To assess BPU on each MBI exam, a quantitative BPU measurement was used, as it was thought to provide an objective assessment and be more sensitive to subtle changes in BPU than could be appreciated with subjective BPU lexicon categories. This quantitative measure was previously found to strongly correlate with radiologist-assigned subjective BPU categories and to have similar associations with breast cancer risk [4]. A single trained operator performed the BPU measurements while blinded to the patient identity and to whether the MBI under analysis was a pre-tamoxifen or post-tamoxifen exam. The quantitative BPU measurement was designed to reflect the lexicon definition of BPU, which refers to the visually assessed intensity of radiotracer uptake in normal breast glandular tissue relative to the intensity of uptake in fat [3]. Thus, the basis of a quantitative BPU measurement on MBI is the ratio of the average pixel intensity in fibroglandular tissue vs. average pixel intensity in fat. The quantitative BPU measure, as previously described [4], is obtained as follows.

Because the functional images obtained with MBI depict the areas of radiotracer uptake but do not distinguish breast anatomy, the most recent mammogram examination from each participant is used to determine fibroglandular and fat regions of interest (ROIs) and these ROIs are applied to the corresponding MBI images. The fibroglandular ROI is first manually drawn on the mammogram to encompass all fibroglandular tissue in the breast and then adjusted by the operator with an intensity threshold to reduce the overall size of the ROI and make it more specific to dense fibroglandular tissue (rejecting skin line, pectoralis muscle, and interspersed areas of less dense tissue and fat). The fat ROI is manually drawn to include a representative area (or areas) of predominantly fat with very little or no dense tissue, outside of the fibroglandular ROI. An outline of the breast is also obtained to assist with the registration of these mammogram ROIs to the MBI. The ROIs, fixed together as a single object, are then copied to the corresponding MBI view and scaled and registered to the MBI to account for the differences in breast positioning and compression. The quantitative BPU measurement for each view is the ratio of the average pixel intensity in the fibroglandular ROI vs. the average pixel intensity in the fat ROI in the MBI image. Values of 1.0 indicate the same intensity of uptake in fibroglandular tissue as compared to fat, values below 1 indicate lower uptake in fibroglandular tissue, and values above 1 indicate higher uptake in fibroglandular tissue.

For the current study, quantitative BPU was measured on both pre-tamoxifen and post-tamoxifen MBI exams on a total of four MBI images, including both right and left breast mediolateral oblique views from both heads of the dual-head gamma camera. A prior analysis showed the quantitative BPU measurement to be similar whether obtained from individual views or an average of combinations of MLO or CC views [4]. In the current study, MLO views were chosen over CC views for analysis as they were observed to more completely visualize the breast fibroglandular tissue and to also provide the pectoralis muscle as a landmark for registering ROIs in the mammography and MBI exams. The same set of ROIs determined from mammograms was used for both pre-tamoxifen and post-tamoxifen exams, with slight adjustments in the position as necessary to register the ROIs to each MBI. An average of these four images was used as a per-patient measure of quantitative BPU at each MBI exam, and this average measure was used for all analyses herein.

### Reproducibility of quantitative BPU

A prior analysis showed the quantitative BPU measurement to be reproducible, with high interoperator and intraoperator agreement (intraclass correlation coefficients [ICC] of 0.92 and 0.98, respectively) [4]. To assess test-retest reliability of quantitative BPU in this study, ROI analysis of all MBI exams was repeated by the same operator in a separate reading session 4 months later, identical to the manner described above, with blinding to the original BPU results. Intraoperator agreement between the original and repeated measurements, assessed by ICC, was 0.96 for pre-tamoxifen MBI exams and 0.98 for post-tamoxifen MBI exams. For each MBI exam, BPU obtained on repeated measurements differed from that on original measurements by an average percent difference of − 2.0% (median 0.9%; sd 6.8%; range − 15 to 11%).

### Analysis

Characteristics of participants taking 5 mg tamoxifen and 10 mg tamoxifen were compared with Student’s *t* test for independent samples or Fisher’s exact probability test, as appropriate. Quantitative BPU measurements on pre-tamoxifen MBI vs. post-tamoxifen MBI were compared with a paired *t* test. Percent change of post-tamoxifen BPU relative to pre-tamoxifen BPU was calculated according to the following equation:

$$ \%\mathrm{change}=\frac{{\mathrm{BPU}}_{\mathrm{post}-\mathrm{tam}}-{\mathrm{BPU}}_{\mathrm{pre}-\mathrm{tam}}}{{\mathrm{BPU}}_{\mathrm{pre}-\mathrm{tam}}} $$

Studies of mammographic density have used a change of at least 5% in magnitude to determine a change in breast response to endocrine therapy [27, 28]. Based on the test-retest reliability results for the quantitative BPU measurement, we selected a change of at least 15% in magnitude to describe a decrease or increase in BPU with tamoxifen for individual participants. A percent change in quantitative BPU within ± 15% was considered unchanged with tamoxifen. The change in BPU from pre-tamoxifen MBI to post-tamoxifen MBI obtained with repeated BPU measurements showed high agreement with that obtained with original BPU measurements (ICC = 0.93); therefore, original measures were used for the primary analysis. Body mass index and mammographic density were considered as confounding predictors of percent change in quantitative BPU and were not significant (*p* >  0.1) and therefore not included in the primary analysis.

## Results

### Participants

Of 47 women invited to the study by letter, 32 (68%) responded to the invitation. After discussing the study procedures with the respondents, 22 of 32 (69%) enrolled in the study, 8 of 32 (25%) respondents declined participation, and the remaining 2 respondents were determined ineligible due to current breast symptoms. Of the 8 respondents who declined participation, 5 cited concerns about the effects of tamoxifen, 1 was unable to travel to the study site for 2 visits, and 2 did not give a specific reason for declining.

Of 22 women enrolled, 21 completed the study and were considered in the analysis, including 10 women prescribed 5 mg tamoxifen per day and 11 women prescribed 10 mg tamoxifen per day. The 1 participant who did not complete the study had initiated 5 mg tamoxifen but withdrew from the study after a lesion was detected on her pre-tamoxifen MBI exam; biopsy of this lesion demonstrated benign pseudoangiomatous stromal hyperplasia.

Participant characteristics are given in Table 1. On average, most participants were in their late 40s (average age 47.7 years), premenopausal (66%), had dense breasts of BI-RADS category c or d (90%), and all were White race. Of 14 premenopausal women, 7 were noted to have pre-tamoxifen and post-tamoxifen MBI performed during the same menstrual cycle phase, either follicular or luteal, with an average difference in cycle day at each imaging point of 3.6 days. The other 7 premenopausal participants did not have cycle information available as 4 had a history of hysterectomy and 3 had a cessation of periods with the placement of an intrauterine device. Approximately 30% of participants had a Gail Model 5-year risk score of at least 1.67%, which was the threshold used for entry into the NSABP P-1 study of tamoxifen for breast cancer prevention. No significant differences in characteristics were observed between the 5 mg group and 10 mg group.

**Table 1:** Participant characteristics

Characteristic*	All analyzable participants (*n* = 21)	Women taking 5 mg tamoxifen (*n* = 10)	Women taking 10 mg tamoxifen (*n* = 11)	*p* (5 mg vs. 10 mg)
Mean age (SD, range)	47.7 (4.1, 41–56)	47.3 (4.4, 41–56)	48.1 (4.0, 42–55)	0.67
Mean body mass index (SD, range)	26.5 (5.1, 19.5–42.4)	26.1 (4.0, 20.7–34.8)	26.8 (6.2, 19.5–42.4)	0.75
Menopausal status**				> 0.99
Premenopausal	14 (66)	7 (70)	7 (64)	
Perimenopausal	6 (29)	3 (30)	3 (27)	
Postmenopausal	1 (5)	0 (0)	1 (9)	
BI-RADS mammographic density***				0.06
a	0 (0)	0 (0)	0 (0)	
b	2 (10)	1 (10)	1 (9)	
c	12 (57)	8 (80)	4 (36)	
d	7 (33)	1 (10)	6 (55)	
Gail model 5-year risk				> 0.99
< 1.67%	15 (71)	7 (70)	8 (73)	
≥ 1.67%	6 (29)	3 (30)	3 (27)	

*Unless otherwise indicated, numbers in parentheses are column percentages for each characteristic, and percentages are rounded

**Menopausal status compared as combined perimenopausal/postmenopausal vs. premenopausal category

***Mammographic density compared as combined b/c category vs. d category

### Participant compliance

The time between pre-tamoxifen MBI and post-tamoxifen MBI was on average 30.3 days (sd = 1.2 days, range 28–33 days). Most of the 21 participants (16 [76%]) completed 30 days of tamoxifen prior to the post-tamoxifen MBI exam, while 4 (19%) completed 29 days and 1 (5%) completed 28 days. All except 1 participant took tamoxifen every day during the study period; the one participant missed a single dose midway through the 30 days.

No participants withdrew from the study due to side effects. Of 21 participants, 8 (38%) reported mild vasomotor symptoms of “hot flashes” and/or “night sweats” experienced at some time during the 30 days of tamoxifen, including 3 of 10 women (30%) on 5 mg tamoxifen and 5 of 11 women (45%) on 10 mg tamoxifen. Four of the 8 participants reporting vasomotor symptoms noted that they had been experiencing these symptoms before enrolling in the study.

### Change in BPU

Quantitative BPU measurements and their changes with tamoxifen are given in Table 2. On the pre-tamoxifen MBI exams, average BPU among participants was 1.43 overall and was not significantly different between women taking 5 mg and 10 mg tamoxifen (1.53 vs. 1.34, *p* = 0.17). BPU measured on pre-tamoxifen and post-tamoxifen MBI did not differ overall (*p* = 0.11). The average change in BPU from pre-tamoxifen to post-tamoxifen MBI was − 6% (sd 17%) but ranged from − 31 to + 45%. Of 21 participants, 7 (33%) showed a decline in BPU (greater than 15% reduction), 12 (57%) did not change BPU (change was within ± 15%), and 2 (10%) showed an increase in BPU (more than 15% increase) after tamoxifen.

**Table 2:** Comparison of background parenchymal uptake, assessed quantitatively, on pre-tamoxifen and post-tamoxifen MBI

	Quantitative BPU on pre-tamoxifen MBI, mean (sd, range)	Quantitative BPU on post-tamoxifen MBI, mean (sd, range)	% change, mean (sd, range)	*p*
Overall (*n* = 21)	1.43 (0.32, 0.81–2.17)	1.34 (0.38, 0.74–2.10)	− 6.1% (17%, − 31 to + 45%)	0.11
Women taking 5 mg tamoxifen (*n* = 10)	1.53 (0.30, 1.12–2.17)	1.51 (0.34, 1.13–2.10)	0.0% (21%, − 24.0 to + 45%)	0.99
Women taking 10 mg tamoxifen (*n* = 11)	1.34 (0.32, 0.81–2.13)	1.19 (0.35, 0.74–1.75)	− 12% (11%, − 31 to + 7%)	0.005

*BPU* background parenchymal uptake, *MBI* molecular breast imaging

**Fig. 1:**
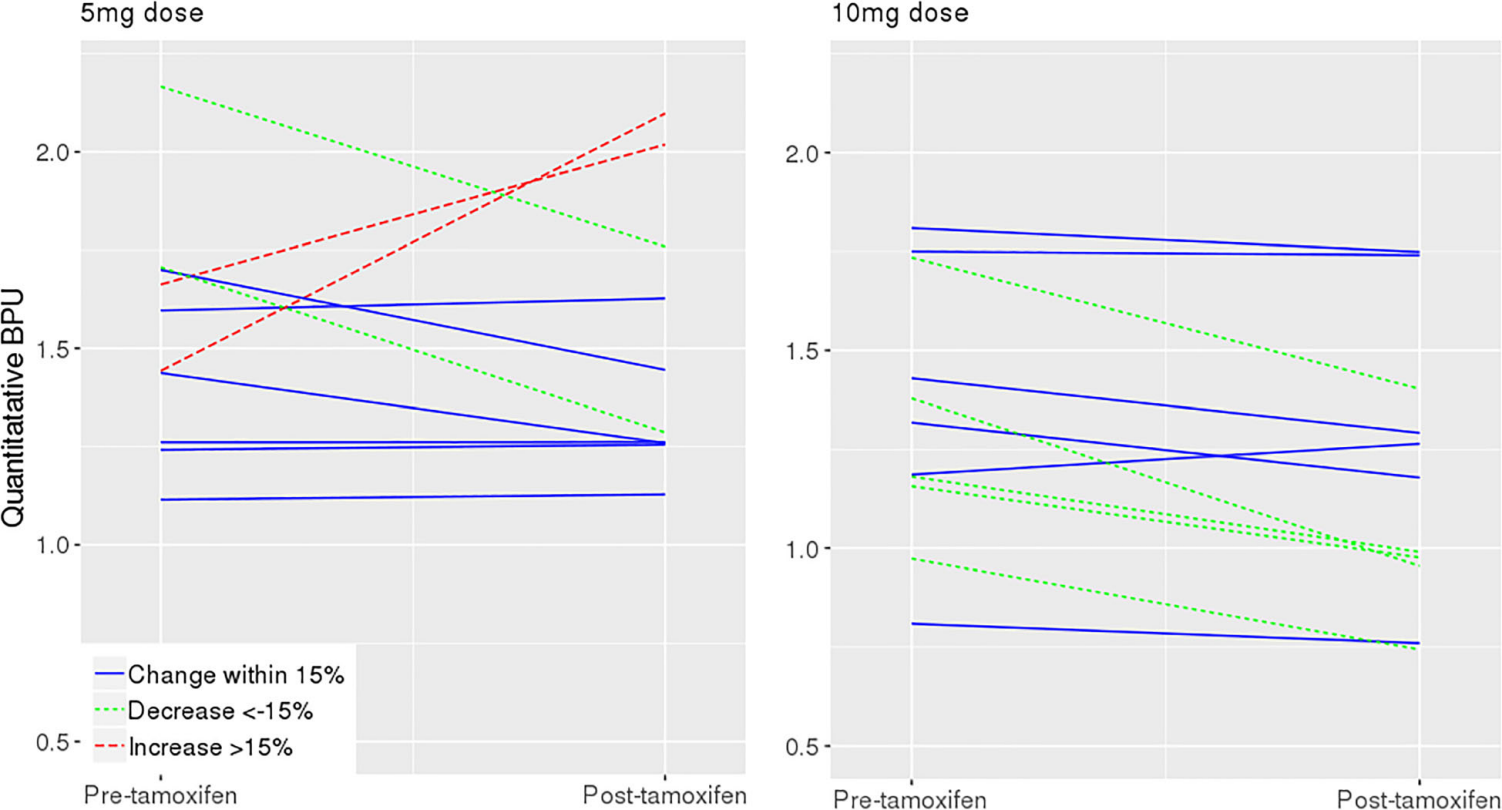
Quantitative BPU measured on pre-tamoxifen MBI and post-tamoxifen MBI for women taking 5 mg tamoxifen per day and 10 mg tamoxifen per day for 30 days

**Fig. 2:**
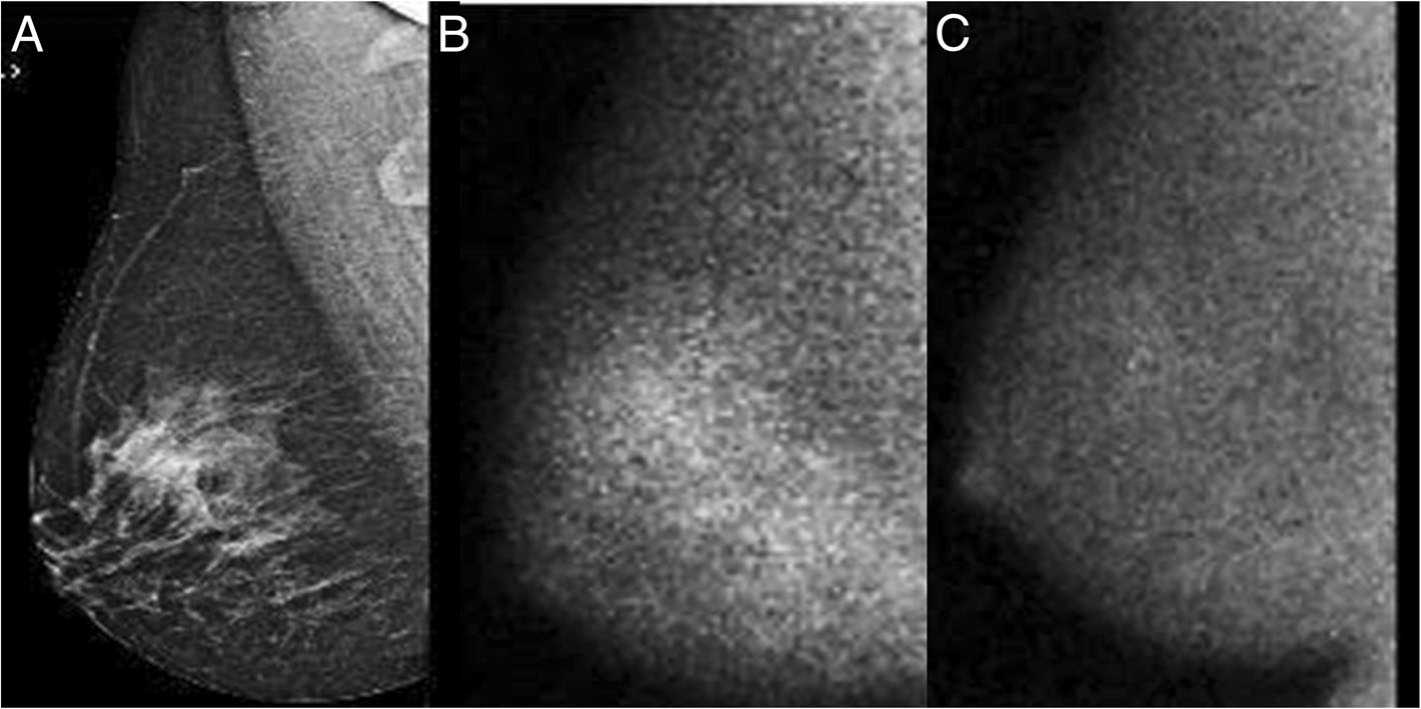
Mediolateral oblique views of the right breast from mammography (**a**) and MBI (**b**, **c**) obtained in a premenopausal study participant taking 5 mg tamoxifen per day for 30 days. The pre-tamoxifen MBI image (**b**) obtained on the first day of her menstrual cycle shows moderate background parenchymal uptake (BPU) with quantitative BPU of 1.9, corresponding to the areas of fibroglandular tissue on the mammogram. The post-tamoxifen MBI image (**c**), also obtained on the first day of menstrual cycle, shows minimal to mild BPU, with quantitative BPU of 1.3, a 31% decline from the pre-tamoxifen MBI

Among the subset of women taking 10 mg tamoxifen per day, BPU on post-tamoxifen MBI was lower than BPU on pre-tamoxifen MBI (*p* = 0.005). However, BPU did not differ between pre-tamoxifen and post-tamoxifen MBI in the subset of women taking 5 mg/day (*p* = 0.99). BPU changes stratified by dose regimen are shown in Fig. 1. In 11 women taking 10 mg tamoxifen, BPU declined in 5 (45%), stayed the same in 6 (54%), and increased in none. In 10 participants taking 5 mg tamoxifen per day, 2 (20%) showed a decline in BPU, 6 (60%) did not change BPU, and 2 (20%) increased BPU after tamoxifen. Example images from a patient showing a decline in BPU with tamoxifen are shown in Fig. 2.

## Discussion

We present the first prospective investigation into whether a short-term intervention of 30 days low-dose tamoxifen could be used to reduce high BPU on MBI. This study provided important information about the feasibility of offering low-dose tamoxifen to women with a history of high BPU on MBI. Although an extensive list of eligibility criteria was employed in this study to ensure safe prescribing of tamoxifen, we observed a relatively high rate of interest from eligible patients, with nearly half of invited women ultimately enrolling in the study. We also observed a high rate of compliance by participants. Although some vasomotor side effects were reported, all were described as mild in severity.

An overall change in average BPU with tamoxifen was not observed; however, reductions were noted in the subset of women taking 10 mg tamoxifen. Among women taking 5 mg tamoxifen, no change in average BPU was observed. These findings suggest a dose-response relationship which leads us to hypothesize that higher tamoxifen doses, such as 20 mg, might lead to a greater decline in BPU; however, low-dose tamoxifen is a preferred approach for minimizing potential side effects. A recently reported study in which women with surgically treated hormone-sensitive ductal carcinoma in situ (DCIS) or lobular carcinoma in situ (LCIS) received 3 years of tamoxifen or placebo demonstrated that tamoxifen at 5 mg/day reduced breast cancer recurrence by half. Importantly, minimal toxicity was observed at this low dose. These latest results have important implications that may impact recommendations for preventive therapy. It may be that the 30-day duration of therapy in our study was inadequate to achieve a significant reduction in BPU at the 5 mg dose [29].

Thus, an alternative to increasing dose may be the longer duration in order to fully observe potential declines in BPU. Prior studies have determined tamoxifen’s half-life as 7 days and estimated that a dosing period of 4 to 6 weeks may be required for tamoxifen to reach steady state [30, 31]. An alternative strategy to arrive at steady state within a 4-week period may be to provide a 1-day loading dose of 20 mg, followed by low-dose tamoxifen for the remainder of the intervention as done in prior studies [32–34]. Future studies may also explore whether other antiestrogen drugs, such as raloxifene, would impact BPU and offer a pharmacokinetic profile better suited to a 30-day administration.

We observed substantial variability in BPU response to tamoxifen among participants, with a range in quantitative BPU change from − 31 to + 45%. This variability in BPU response may reflect the known variability in response or resistance to tamoxifen among patients, which has been explained by several factors, including differing estrogen receptor function and genetic polymorphisms of drug-metabolizing enzymes. Studies examining one of these enzymes, CYP2D6, have yielded conflicting results regarding its ability to predict tamoxifen’s efficacy in chemoprevention [35]. The controversy stems from two factors: (1) by relying on tumor samples to derive the CYP2D6 genotype, some studies had genotyping errors and (2) enzymes other than CYP2D6 are involved in the complex metabolism of tamoxifen [36]. Thus, genetic testing is not routinely recommended before initiating chemoprevention. An imaging biomarker that reflects tissue response to tamoxifen, or more specifically the active metabolite endoxifen, rather than enzyme genotype may be a more accurate marker of chemoprevention effectiveness. The investigation into associations of imaging response to tamoxifen and genotyping results is needed.

It is possible that the observed variation in BPU response was impacted by other factors beyond tamoxifen. Although we excluded women taking exogenous hormones, women may have had changes in BPU due to fluctuations in endogenous hormones. In a prior study that imaged premenopausal women at both peak follicular and peak luteal phase with MBI, most women did not show a change in BPU with cycle phase, but in the 29% who did, BPU was always higher in the luteal phase [9]. Although logistics of study scheduling prevented precise timing of MBI exams with the menstrual cycle, the 30-day period between scans ensured that participants with regular cycles (*n* = 7) were imaged at approximately the same time in the cycle phase, either follicular or luteal for pre-tamoxifen and post-tamoxifen MBI. However, in seven other premenopausal participants and six perimenopausal participants, long cycles or lack of cycles prevented the verification of MBI timing with the cycle.

Two subjects showed an increase in BPU with tamoxifen, which was unexpected. We were unable to assess whether this increase could be an artifact of endogenous hormonal changes, due to the lack of recent menstrual cycle information (one perimenopausal and one with cycle cessation due to IUD placement). Another potential explanation could be the existence of a metabolic flare reaction, which refers to an initial increase in radiotracer uptake with treatment. In a study of breast cancer patients undergoing treatment with tamoxifen, metabolic flare was observed in breast tumors and metastatic disease sites on positron emission tomography (PET) with glucose analog F-18 fluorodeoxyglucose (FDG); an early increase in FDG uptake after 7 to 10 days of tamoxifen was predictive of ultimate response to tamoxifen [37]. However, there is no evidence to date to establish whether a similar metabolic flare can occur with Tc-99m sestamibi uptake in non-malignant breast tissue.

As a pilot study, we had a limited sample size of mostly young and premenopausal or perimenopausal women; thus, our results may not be generalizable to postmenopausal women. However, these findings are important as tamoxifen is the first-line therapy in adjuvant and chemoprevention settings for women who have yet to go through menopause. This study did not include a control group receiving a placebo drug; doing so may have allowed for better evaluation of side effects attributable to the tamoxifen and comparison of declines in BPU with controls. Participants were not randomized to 5 mg or 10 mg tamoxifen (first 10 enrolled received 5 mg and subsequent participants received 10 mg), but the groups were generally similar in composition. A higher number of women in the 10 mg group had the highest category of mammographic density, extremely dense, although not significantly different from the 5 mg group. Despite a potential difference in density, the pre-tamoxifen quantitative BPU of the 5 mg and 10 mg groups did not significantly differ.

Our work evaluating BPU has several parallels to investigations of background parenchymal enhancement (BPE) on MR imaging. BPE, which describes the amount of gadolinium enhancement in non-malignant breast tissue, has also been shown to vary among women with similar mammographic density and similar MR-depicted fibroglandular tissue [19]. High BPE has been associated with both prevalent breast cancer and risk of incident breast cancer [19, 38, 39]. Like BPU, BPE is influenced by hormonal factors such as menopausal status and menstrual cycle [40, 41]. BPE has been shown to decrease with the use of tamoxifen and aromatase inhibitors [20, 42]. Together, this evidence speaks to the potential of functional imaging techniques of MR imaging and MBI to be useful in evaluating normal fibroglandular tissue of the breast in order to understand normal hormonally influenced variants and indicators of potential breast cancer risk.

Findings from this pilot study justify future studies evaluating the use of functional imaging, and MBI in particular, to assess the efficacy of a chemopreventive treatment such as tamoxifen. Although BPU has been established as a marker of breast cancer risk, it will be important to determine if the reduction in BPU is indicative of a concomitant reduction in breast cancer risk.

## Conclusions

In summary, short-term intervention with low-dose tamoxifen was well-tolerated by study participants and was associated with a reduction in BPU for some women. Our results suggest that 10 mg tamoxifen per day is more effective than 5 mg/day for reducing BPU. Given the variability in BPU response to tamoxifen observed among study participants, future study is warranted to determine if BPU response could be used to predict the effectiveness of tamoxifen for breast cancer risk reduction.

## Abbreviations

BI-RADS: Breast Imaging-Reporting and Data System
BPU: Background parenchymal uptake
MBI: Molecular breast imaging
MR: Magnetic resonance
ROI: Region of interest
